# MiRNAs Targeting Double Strand DNA Repair Pathways Lurk in Genomically Unstable Rare Fragile Sites and Determine Cancer Outcomes

**DOI:** 10.3390/cancers12040876

**Published:** 2020-04-03

**Authors:** Stephan Marquardt, Christin Richter, Brigitte M. Pützer, Stella Logotheti

**Affiliations:** 1 Institute of Experimental Gene Therapy and Cancer Research, Rostock University Medical Center, 18057 Rostock, Germany; stephan.marquardt@med.uni-rostock.de (S.M.); christin.richter@med.uni-rostock.de (C.R.); 2 Department Life, Light & Matter, University of Rostock, 18059 Rostock, Germany

**Keywords:** rare fragile sites, FRAXA, cancer, miRNA, non-homologous end-joining, homologous recombination, double-strand DNA repair

## Abstract

Double strand break (DSB) repair mechanisms guard genome integrity and their deterioration causes genomic instability. Common and rare fragile sites (CFS and RFS, respectively) are particularly vulnerable to instability, and there is an inverse correlation between fragile site (FS) expression and DSB repair protein levels. Upon DSB repair dysfunction, genes residing at these sites are at greater risk of deregulation compared to genes located at non-FS. In this regard, it remains enigmatic why the incidence of miRNA genes at FS is higher compared to non-FS. Herein, using bioinformatics, we examined whether miRNA genes localized at FS inhibit components of DSB repair pathways and assessed their effects on cancer. We show that such miRNAs over-accumulate in RFS, and that FRAXA, which is expressed in Fragile X syndrome, is a conserved hotspot for miRNAs inhibiting DSB repair. Axes of FRAXA-residing miRNAs/DSB repair targets affect survival in a cancer type-specific manner. Moreover, copy number variations in the region encompassing these miRNA genes discriminate survival between male and female patients. Given that, thus far, only CFS have been considered relevant for carcinogenesis, our data are the first to associate RFS with cancer, through the impairment of DSB repair by the FRAXA-residing miRNAs.

## 1. Introduction

Each human cell is subjected to a daily barrage of DNA assaults that generate either single-strand DNA (ssDNA) or double-strand DNA breaks (DSBs). To cope with this, cells use a variety of DNA repair mechanisms. The less frequent but more deleterious DSBs are repaired via two distinct mechanisms, homologous recombination (HR) and non-homologous end joining (NHEJ), each one encompassing distinct components and predominating during different phases of the cell cycle (Table 1). HR repairs DSBs by utilizing equivalent regions of DNA between homologous or sister chromosomes as templates, whereas NHEJ relegates the ends independently of a template [1]. If HR and/or NHEJ perform inaccurately, they become error-prone and thereby propel genomic instability through slipping to alternative types of end joining (alt-EJ) and DSB repair [2].

Some conserved genomic regions, the so-called fragile sites (FS), are less stable than others, meaning that genomic instability at these loci precedes the one in the other genomic regions [37]. Fragile sites are inherently difficult to replicate, so that even low doses of replication inhibitors lead to stalling and collapse of the replication forks. As a result, FS exhibit gaps, breaks or rearrangements on metaphase chromosomes, which are collectively referred to as FS expression [38]. Due to this unstable genomic environment, genes residing within or in proximity to FS are at greater risk of deregulation upon FS expression, compared to genes located at non-fragile sites [39]. Deletions of tumor suppressor genes and amplifications of oncogenes, for instance, can occur upon breakage at FS [39]. Genes residing in FS include both, protein-coding and non-coding RNA genes, such as miRNAs [40]. Based on previous reports, a significant number of miRNA genes are located at FS as compared to those located at non-fragile regions [41,42], although the necessity for this genetic arrangement remains unknown. The integrity of the FS loci relies largely on ATM and/or ATR kinases [43,44,45] and the downstream DSB repair pathways [46]. The perturbation of DNA replication leads to the formation of DSBs at FS, which are repaired by HR and NHEJ. Upon downregulation of components of either NHEJ or HR, FS expression increases. Consequently, FS expression is inversely correlated with the levels of NHEJ or HR components [46].

Depending on their frequency within the human population, FS are classified into common fragile sites (CFS) or rare fragile sites (RFS), with each class exhibiting distinct characteristics. On the one hand, CFS are abundant in the genome, encountered in every individual, induced by aphidicolin (APH), bromodeoxyuridine (BrdU) or 5-azacytidine (5-aza) and considered to be an intrinsic part of the chromosomal structure. They associate with deletion and translocation breakpoints, constitute sites of integration for oncogenic viruses and gene amplification in tumor cells, and as such, they are considered hotspots of carcinogenesis [47]. All CFS examined to date are comprised of AT-rich flexibility islands, which hinder replication, especially under conditions of replication stress. This may result in long stretches of ssDNA that are able to form stable secondary structures such as hairpins or cruciforms, which further stall replication fork progression, thereby triggering an ATR repair pathway. Defective ATR pathway signaling could result in DNA breakage at these sites, leading to cancer-specific chromosomal aberrations [47].

On the other hand, RFS are fewer than CFS across the genome, inherited in a Mendelian manner and are found in less than five percent of human population. Most RFS are expressed under folate-deficient conditions, whereas others are induced by chemicals that bind AT-rich DNA such as distamycin A or Berenil. Fragility at rare sites is attributed to the expansion of either a CGG repeat or an AT-rich minisatellite, which can also form stable secondary structures [39]. Clinically, RFS are mainly associated with mental retardation [48] and bipolar disorder [49].

Common fragile sites can be found in all healthy individuals and are frequently expressed in cancer. In contrast, RFS expression occurs only within a small percentage of the population and has not yet been associated with increased cancer risk [48]. However, RFS share with CFS the same mechanistic basis of fragility, because they both adapt stable non-B DNA structures that act as physical barriers to replication forks [50]. This may in turn result in the collapse of replication forks followed by DNA breakage and dysregulation of genes hosted there [49]. In this regard, even if RFS are not hereditarily expressed in healthy non-carriers, they can theoretically be expressed in their somatic cells, if DSB repair machineries are compromised, eventually leading to the deregulation of RFS-hosted genes. Implications of such perturbations in terms of cancer remain unknown, however, deregulation of RFS-residing genes have been recently shown to affect invasiveness. For instance, *FMR1*, a FRAXA-residing gene which harbors trinucleotide repeat expansions in Fragile X syndrome (FXS) patients, correlates with aggressiveness and lung metastasis probability in triple-negative breast cancer. Overexpression of the FMR1 protein enhances, whereas its downregulation inhibits breast cancer metastasis [51]. Links between *FMR1* and other cancer types have been recently reported [52]. In addition, *MIR888*, also located at the Xq27.3 region which includes the FRAXA site, is upregulated across several cancer types to orchestrate invasive tumor growth. We have recently shown that this is achieved via the miR-888-mediated inhibition of NHEJ component APLF, causing impairment of the NHEJ machinery, which exacerbates genomic instability and promotes invasiveness in a cancer type-specific manner [53]. The abovementioned gene deregulations can be indicative of alterations in the corresponding chromosomal regions and highlight the need to investigate underlying associations between RFS-residing genes and cancer. Moreover, based on the fact that invasiveness can be achieved through the upregulation of at least one miRNA gene within the FRAXA region with the ability to perturb DSB repair machinery [53], we were wondering if RFS-hosted miRNA genes are systemically able to affect cancer outcomes through involvement in DSB repair pathways.

## 2. Results and Discussion

### 2.1. RFS Presents a Higher Density of miRNA Genes Compared to CFS

**Figure 1:**
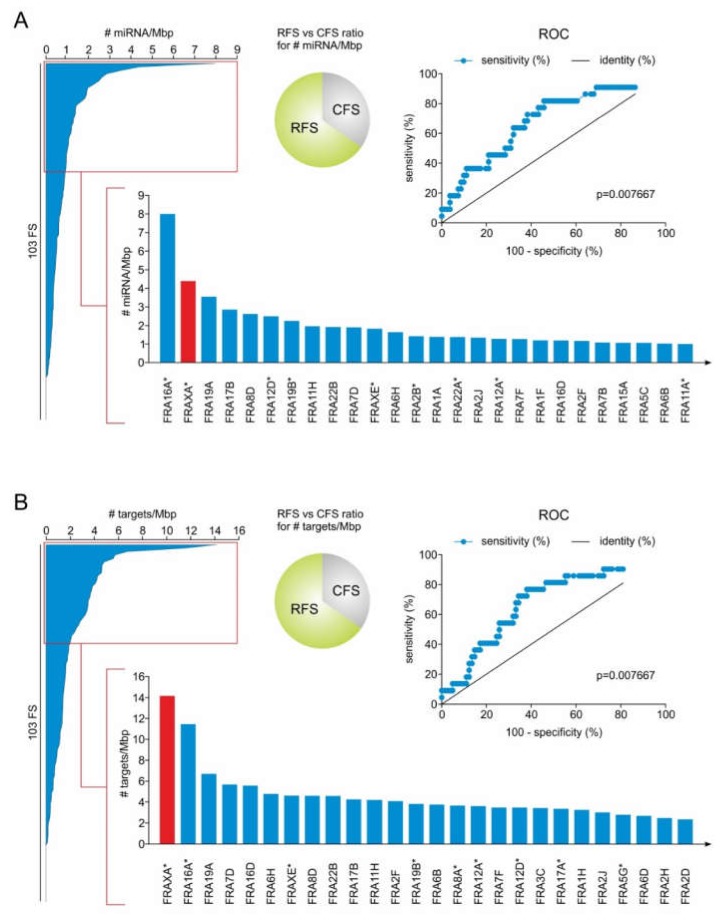
Ranking of FS according to density of miRNA genes (miRNA/Mbp) and DSB repair genes targeted by those miRNAs (targets/Mbp). RFS have 1.9 times the miRNA (**A**) and target (**B**) density compared to CFS (each top center) and rank on top regarding both characteristics (see ROC curves and bar graph at the bottom). RFS sites are denoted with an asterisk.

The cytogenetically defined CFS and RFS and their corresponding chromosomal regions have been published previously [54,55,56,57,58] and were updated based on the latest NCBI gene entries. MiRNA genes located at the regions of these CFS and RFS were identified [40] and extracted as described in Materials and Methods (Supplementary Table S1), and their density, expressed as number of miRNAs per mega base pairs (Mbp) of FS, was determined for each FS. As shown in Figure 1A, miRNA genes are not evenly distributed, but tend to over-accumulate in specific FS. In particular, only 21% of all FS (22 out of 103) are RFS, but they present the highest density in miRNA genes, with 8 of them ranking among the top 20 FS that are most populated in miRNA genes. Collectively, among all FS, the RFS FRA16A and FRAXA are ranked on top having the highest density of miRNA genes (Figure 1A bottom, Supplementary Table S2).

In contrast, the FS with the lowest density in miRNA genes include the CFS FRA4B, FRA7K, FRA6F, FRA4F and FRA5F. In terms of miRNA gene expression, a single breakage in a RFS with the highest miRNA gene density is more likely to deregulate the miRNA genes, as compared to FS with low miRNA gene density. This means that a lesion in the top-ranked FRA16A and FRAXA stands higher chances to affect expression of the corresponding miRNA genes as compared to several frequently expressed CFS, such as FRA3B and FRA6E [59], which rank at the bottom of the list (Supplementary Table S2).

### 2.2. MiRNA Genes Targeting DSB Repair Components Over-Accumulate in RFS

Upon FS expression, the hosted miRNA genes can be deregulated. If an expressed FS hosts miRNA genes with the ability to inhibit components of the DSB repair pathways post-transcriptionally, then their subsequent deregulation could perturb the expression of these components, ultimately affecting DSB repair. To this end, we examined whether FS host miRNA genes that putatively target components of HR and NHEJ machinery, as well as their crucial upstream regulators (see Table 1). The potential targets of the FS-residing miRNAs were retrieved from the microT-CDS DIANA database v5.0 [60,61] and their targeting tendency was estimated as number of all DSB repair genes targeted by miRNAs of each FS normalized to the length of each FS. Here, we found that 40% of the top-20 FS with the highest number of DSB repair targets are RFS, despite the fact that they constitute only 21% of total FS. RFS were found to be significantly enriched in miRNA genes targeting components of DSB repair pathways compared to CFS. Interestingly, the RFS FRAXA and FRA16A were clearly ranked among the top FS, which are overpopulated by miRNA genes that inhibit DSB repair targets (Figure 1B bottom, Supplementary Table S3). Notably, FRAXA emerged as the FS with a high density of miRNA genes that can potentially target the highest number of DSB repair factors per Mbp, thereby supporting a functional role of the FRAXA-located miRNA genes in these pathways. Hence, we turned our attention to this specific FS.

### 2.3. FRAXA is a Conserved Hotspot for miRNA Genes Inhibiting Components of DSB Repair Pathways

**Figure 2:**
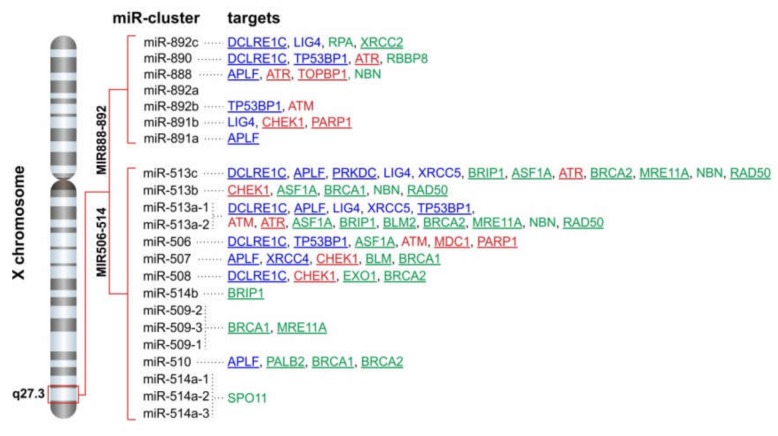
FRAXA is a hotspot of miRNA genes targeting DSB repair. The two microRNA clusters MIR506-514 and MIR888-892 are located at X chromosomal band q27.3, upstream the FMR1 gene. Potential target genes of single miRNAs are shown (blue: non-homologous end-joining; green: homologous recombination; red: upstream regulators). Targets with an impact on survival based on the PanCan data are underlined.

The chromosomal band Xq27.3, where FRAXA resides, hosts 22 miRNA genes. We found that 15 of these microRNAs have the ability to potentially target one or more DSB repair pathway components, and overall, we calculated 71 individual hits on DSB factors (Figure 2). The FRAXA-related miRNA genes are grouped in two distinct clusters: the MIR888-892 and the MIR506-514 cluster. Both clusters encompass rapidly evolving primate-specific miRNA genes, with testis-specific expression, and have been correlated with male sexual maturation, testis development and spermatogenesis [62,63,64,65].

**Figure 3:**
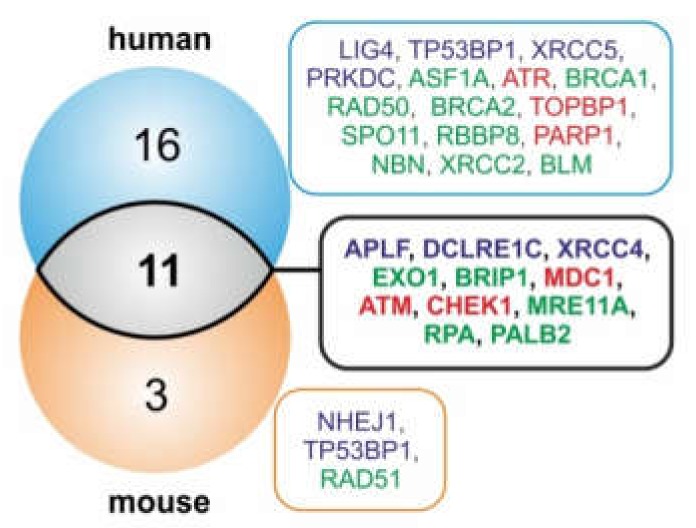
Mouse and human FX-residing miRNA genes share common DSB repair pathway targets, and are independent of miRNA gene sequence homology. Blue: non-homologous end-joining; green: homologous recombination, red: upstream factors.

It has been recently shown that pre-primates also host a miRNA cluster in the Fragile-X region of the X chromosome, with a conserved position and orientation relative to neighboring genes. A comparison between mouse and human FRAXA-miR (FX-miR) clusters showed that, although they have divergent sequences and do not present true gene homologues, they exhibit a conserved ability to inhibit the same gene target *FMR1* and be expressed in the same cell types. This can be achieved through evolutionary conservation of their position in cis and upstream to *FMR1*. This study provided the first evidence that the function of these miRNAs is determined through the conservation of their location in the genome, irrespective of their primary sequence [66]. This intriguingly suggests that a functional conservation of these miRNA genes could be achieved through their positional conservation, a notion that has recently been suggested for long non-coding RNA genes [67]. With this in mind, we further wondered whether the putative DSB repair targets of the FX-miRs are also conserved in mice. Indeed, using microT-CDS tool [60,61], we predicted that miRNAs of the mouse FX-miR cluster target components of both NHEJ and HR repair machineries (Supplementary Table S4). The tendency to potentially target the components APLF, DCLRE1C, XRCC4 (NHEJ), BRIP1, MRE11A, PALB2, RPA1, EXO1 (HR), and ATM, CHEK1, MDC1 (upstream regulators) is conserved between the human and the mouse FX-miR clusters (Figure 3).

**Figure 4:**
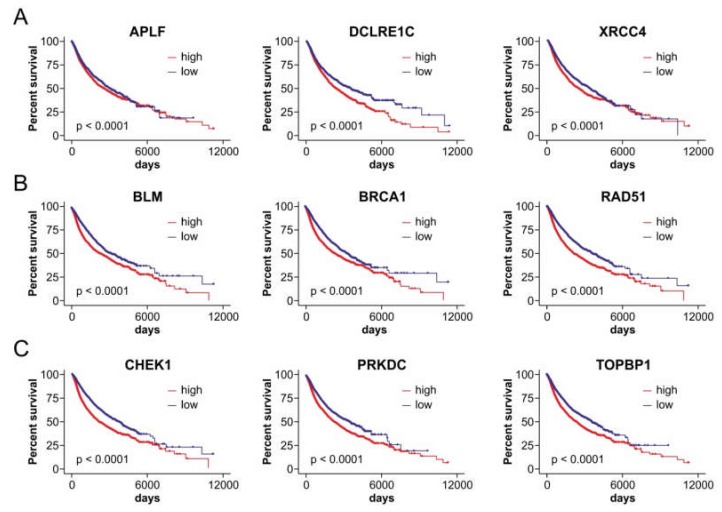
High expression of DSB repair genes targeted by members of FX-miR clusters correlates with worse cancer patient survival. The graphs show Kaplan–Meier curves from PanCan data of representative DSB repair components associated to non-homologous end-joining (**A**), homologous repair (**B**), and upstream targets (**C**) APLF, DCLRE1C, XRCC4, BRCA1, RAD51, CHEK1, and TOPBP1 are conserved targets of FX-miR clusters between mouse and human.

This target conservation could imply a systemic need for miRNA genes that fine-tune these DSB repair factors to preserve the genomic position in Xq27.3, perhaps to ensure activation of the respective miRNA/target axes under certain conditions to which the FRAXA region is responsive, e.g., folate availability. Indeed, a Cox hazard analysis of PanCan patient data revealed that high levels of DSB repair factors which constitute conserved targets of the FX-miRs, such as APLF, DCLRE1C, XRCC4, BRCA1, RAD51, CHEK1, and TOPBP1, correlate with worse cancer patient survival (Figure 4; Supplementary Table S5). This finding is in line with observations that the deregulation of DNA repair is associated with resistance to radiation therapy and cancer progression [68,69,70,71]. In summary, the positionally conserved Xq27.3-residing miRNA genes and their potential DSB repair pathway targets might play a crucial role for cancer progression and outcomes.

### 2.4. Axes of FRAXA-Residing miRNA Genes and Their DSB Repair Targets Affect Patient Outcomes in a Cancer Type-Dependent Manner

The emerging link between Xq27.3-residing miRNA genes and components of DSB repair pathways that significantly affect cancer outcomes indicates an under-noticed implication of this fragile genomic location in cancer via perturbations of the DSB repair machinery. This is further encouraged by the fact that patients with fragile X syndrome, who bear an impaired FRAXA region, exhibit downregulation of DNA damage/repair pathway transcripts [72]. These data support the existence of an inverse correlation between FRAXA expression and levels of DNA repair factors. Such correlations could, at least in part, be mediated by the deregulation of FRAXA-residing, DSB repair-inhibiting miRNA genes, and have an impact on cancer progression. These features, combined with the fact that the FRAXA region is prone to a loss of genomic integrity, e.g., gaps, breaks, rearrangements or translocations [45,73,74], lead to the postulation that an alteration at this particular FS stands increased chances of deregulation of the proximal miRNA genes. This, in turn, might disturb the levels of components of the DSB repair pathways. In the context of cancer, the expression of the corresponding miRNA/DSB repair gene target axes could ultimately affect disease outcomes.

To test this hypothesis, we initially checked whether FX-miRs are found expressed in cancer patients. We kept in mind that FX-miRs are evolutionarily young miRNAs and, as such, they are expressed in few tissues and at low levels [75]. Members of the FX-miRs normally present testis-specific expression patterns [62,63,64,65] and can be under strict epigenetic silencing [53]. Consequently, the off-context activation of the primate-specific members of FX-miR clusters may not only suggest an implication in cancer [53], but also be an indicator of deregulated activity of the Xq27.3 region. Indeed, analyses of the PanCan cohort, which includes gene and miRNA expression data in 32 different cancer types, showed ectopic expression of almost all representatives from both MIR506-514 and MIR888-892 clusters in tissues other than testes. The loss of the tissue-restricted expression patterns suggests deregulation of these miRNA clusters in tumors. For instance, miR-508 (98%) and miR-891a (85% of patients) were expressed across all tissues. Other members of both clusters were also expressed across most tissues. For example, from the MIR506-514 cluster, miR-514 is expressed in 79%, and miR-506 in 42% while expression of the other miRNAs was detected in 10% to 34% of patients. Additionally, members from the MIR888-892 cluster were expressed in 6% to 28% of patients (Supplementary Table S6).

**Figure 5:**
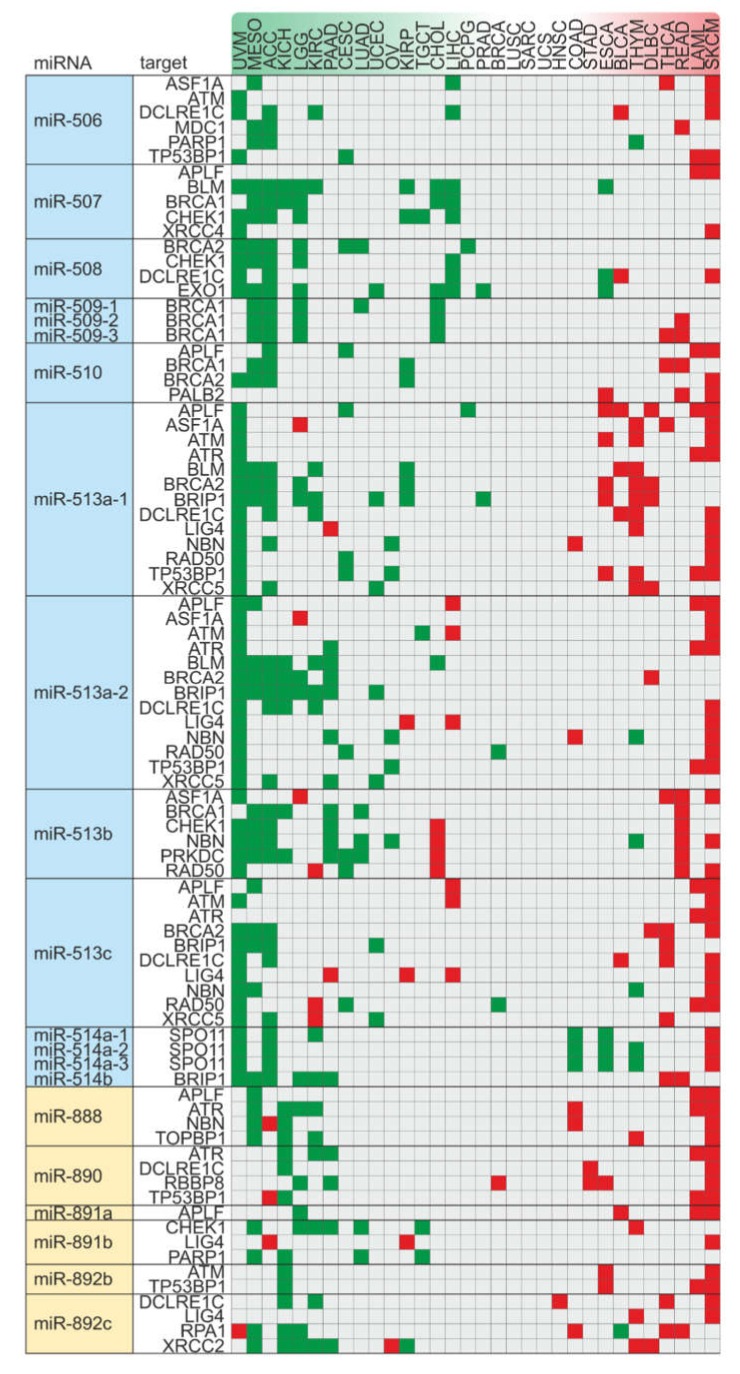
Cancer type-specific effects of axes of FX-miR/DSB repair mRNA target on patient survival. Cancer types are ranked based on the number of axes with an impact, either beneficial (DSB repair target indicating worse prognosis AND miRNA correlates with better prognosis, green) or detrimental (DSB repair target indicating improved prognosis AND miRNA correlates with worse prognosis, red), on disease outcome. Cox analysis *p*-value < 0.1 for targets and miRs. Abbreviations (in alphabetical order): ACC: Adrenocortical carcinoma, BLCA: Bladder Urothelial Carcinoma, BRCA: Breast invasive carcinoma, CHOL: Cholangiocarcinoma, COAD: Colon adenocarcinoma, DLBC: Lymphoid Neoplasm Diffuse Large B-cell Lymphoma, ESCA: Esophageal carcinoma, HNSC: Head and Neck squamous cell carcinoma, KICH: Kidney Chromophobe, KIRC: Kidney renal clear cell carcinoma, KIRP: Kidney renal papillary cell carcinoma, LAML: Acute Myeloid Leukemia, LGG: Brain Lower Grade Glioma, LIHC: Liver hepatocellular carcinoma, LUAD: Lung adenocarcinoma, LUSC: Lung squamous cell carcinoma, MESO: Mesothelioma, OV: Ovarian serous cystadenocarcinoma, PAAD: Pancreatic adenocarcinoma, PCPG: Pheochromocytoma and Paraganglioma, PRAD: Prostate adenocarcinoma, READ: Rectum adenocarcinoma, SARC: Sarcoma, SKCM: Skin Cutaneous Melanoma, STAD: Stomach adenocarcinoma, TGCT: Testicular Germ Cell Tumors, THCA: Thyroid carcinoma, THYM: Thymoma, UCS: Uterine Carcinosarcoma, UCEC: Uterine Corpus Endometrial Carcinoma, UVM: Uveal Melanoma.

Then, to systemically monitor whether the deregulated axes of FX-miRs and their downstream DSB repair mRNA targets associate with cancer outcomes, we meta-analyzed TCGA patient data. Expression correlation analysis between miRNAs and DSB repair factors indicates a cluster-wise activation of these miRNA genes (Supplementary Table S6). We subsequently performed Cox regression analysis to investigate cancer prognosis based on the combined effect of these miRNAs and their corresponding DSB repair targets, for each cancer type. The results for each miR/mRNA target axis across all cancer types are demonstrated in Figure 5 and are ranked based on the number of axes with an impact, either beneficial (more left) or detrimental (more right), on disease outcome. Interestingly, the most affected cancer types include UVM (uveal melanoma), ACC (adrenocortical carcinoma), MESO (mesothelioma), KICH (kidney chromophobe), LGG (lower grade glioma), SKCM (skin cutaneous melanoma), LAML (acute myeloid leukemia) and THYM (thymoma). In ACC, UVM, MESO, KICH and LGG, these axes have mainly a favorable impact on prognosis and include members of the MIR506-514 cluster and their corresponding targets. In contrast, in SKCM, LAML and THYM, these axes are associated with poorer prognoses and include members of both MIR506-514 and MIR888-892 clusters. The list of affected cancer types also includes DLBC (large B-cell lymphoma), READ (rectum adenocarcinoma) and THCA (thyroid carcinoma), where axes of both clusters and associated targets exert an exclusively negative effect on patient outcomes, while in CESC (cervical squamous cell carcinoma and endocervical adenocarcinoma), LUAD (lung adenocarcinoma), and UCEC (uterine corpus endometrial carcinoma) these axes have an exclusively positive influence on survival. Interestingly, sex-specific cancers, such as BRCA (breast invasive carcinoma) in females, and PRAD in males are included among those which remain least-affected, while UCS (uterine carcinosarcoma), SARC (sarcoma) and LUSC (Lung squamous cell carcinoma) are unaffected by these axes. Other cancer types are both positively and negatively affected by FX-miRs and their downstream DSB repair mRNA targets. Moreover, no single miRNA/target or miR cluster/targets axis was universally associated with prognosis across all cancer types. Rather, these axes affect prognosis in a cancer type-dependent manner. These data provide insights on the cancer types where FX-miRNA/DSB repair target axes are clinically meaningful.

Collectively, these results indicate that FX-miRs are ectopically activated in tissues other than testes and that they influence specific cancer types through DSB repair. In addition to DSB repair ([53] and this study), individual FX-miRs have been reported to affect diverse gene targets and cancer-related processes, such as cancer cell proliferation, migration and invasion, epithelial-mesenchymal transition (EMT), chemo- and radiosensitivity (reviewed in Supplementary Table S7). This manifests that, in agreement with our findings, the manner through which FX-miRs influence processes mediating tumor formation and/or progression is highly cancer-type dependent. The observation that neither FX-miRs nor FX-miR/DSB repair targets exert a universal effect on all cancer types or even cancer cells of the same cancer type [76] can be attributed to the fact that mammalian miRNAs rarely exert dominant-type behaviors. The ultimate effect of an individual miRNA on cellular phenotypes is shaped by several factors, mainly (a) the presence of co-operating miRNAs and common downstream mRNA targets in the cell milieu, (b) the levels of upstream transcription factors, and (c) the degree of its interconnection with other components in a network. More than one miRNAs co-regulate a single mRNA target and, vice-versa, a single miRNA can simultaneously inhibit multiple, divergent mRNA targets. Thus, the cellular context in which a particular miRNA is expressed largely influences its behavior. A complex interplay among miRNA and its associating molecules, mainly its upstream transcription factors and downstream mRNA targets, takes place within the cell milieu to determine the actual function in which a miRNA will ultimately be engaged (reviewed in [77]). In this respect, each member of the FX-miR clusters has a distinct set of downstream mRNA targets, can co-operate with divergent miRNAs and be controlled by different transcription factors. Hence, it is not surprising that, even though they reside at the same hotspot, the FX-miRs may exert mixed effects on cancer outcomes. Their behavior can be oncogenic, anti-oncogenic, or Janus, according to both the cancer type and the cell context. There are also cases of FX-miRs, namely miR-514b, where the 3p and 5p strands can have opposing roles in the same cellular process through inhibiting divergent gene targets, even though they are transcribed from the same hairpin, a fact that further warrants for the inconsistent effects of these miRNA genes on cancer [78]. Furthermore, the evolutionary age determines miRNA behavior, since the ones that originated in more distant ancestors had adequate time to develop robust interconnections within the network, co-evolve with crucial targets, and occupy hub positions, while the evolutionarily young miRNAs have been recruited to further assist the function of evolutionarily old miRNAs [77]. FX-miRs are recently evolved and, as such, they are less likely to have predominant impact on disease phenotypes, as opposed to ancient human miRNAs, which are enriched for origins near the dawn of animal multicellularity [79]. Importantly, our analysis provides the first comprehensive evidence that the miRNA genes overpopulating a genomically unstable RFS are involved in cancer through targeting the machinery that ensures genomic stability. Although, thus far, deregulation of FX-miR clusters has not been causatively linked to FRAXA induction, their ectopic expression in cancer is however suggestive of altered transcriptional, epigenetic and/or structural characteristics of the Xq27.3 chromosomal region. The fact that cancer types which are mostly affected by these axes are relatively rare [80,81,82,83] could provide an explanation why such alterations may have gone under-noticed to date. Future proof-of-principle experiments to investigate whether these cancer types present breaks, enhancements or translocations at FRAXA, accompanied by deregulated DNA repair activity, would provide invaluable mechanistic insights on the involvement of the Xq27.3 region in DSB repair. For instance, target validation can be conducted according to established experimental pipelines [53] and the inverse expression correlation of miRNAs and targets could be confirmed by real-time PCR in these cancer types. In parallel, fluorescence in situ hybridization (FISH) experiments and/or copy number variation analysis using probes against the chromosomal region encompassing the FX-miR clusters can be performed and correlated with the miRNA/mRNA ratio. The effect of DNA damaging agents and replication fork inhibitors on the FX-miRs, their corresponding targets and the replication forks activity in the Xq27.3 region could also be estimated and their downstream effects in the activity of the DSB repair machinery could be monitored using relative repair assays. Moreover, given that FRAXA is expressed upon folate-deficiency, it would be interesting to investigate whether folate treatment can modify DSB repair activity and cancer cell phenotypes via preservation of the integrity of Xq27.3 region and the FX-miR/DSB repair target ratio. These interactions can complementarily be investigated in cells where FRAXA is expressed, i.e., cells of FX syndrome patients, as well as in normal sites of FX-miR expression, i.e., in testis tissues and in spermatogenesis in vitro and in vivo models. Such experiments will comprehensively unveil the mechanism of interplay among Xq27.3 region, FX-miR expression and the DSB repair machinery. Furthermore, from the translational point of view, such studies could also offer novel perspectives towards the advancement of diagnostic and/or therapeutic strategies for cancer types, the rarity of which poses as a challenge in their personalized management.

Our results underscore a newly-identified association between DSB repair and the FRAXA-localized miRNAs. The residing of miRNA genes that jeopardize genomic stability in a FS sounds paradoxical. We propose that such a genomic arrangement can serve as a hypersensitive sensor of proper functionality of the DSB repair machinery, at least under conditions of folate stress. In detail, folate deficiency causes replication-associated DNA breakage and wide-spread genomic instability, and FRAXA belongs to the category of RFS which are particularly sensitive to folate deprivation, especially when CGG trinucleotide repeats in the *FMR1* gene expand beyond a critical size. Characteristics of FRAXA site that contribute to its instability are proposed to be (a) atypical DNA structures formed by the CGG repeat itself and (b) defective mitotic sister chromatid disjunction of genomic regions containing long CGG repeats [73]. The existence of FX-miR clusters that target DSB repair pathways upstream of the site of formation of long CGG repeats provides new insights on how genome instability could be spread. If a breakage in FRAXA, i.e., under conditions of folate deficiency, can indeed activate the FX-miR clusters, then the subsequent inhibition of both HR and NHEJ pathways could lead to “crashing” of the DSB repair machinery and, eventually, apoptosis. Such a model would theoretically favor the elimination of cells with suboptimal DSB repair functionality from the rest of the cell population in cells with functional apoptotic pathways downstream DSB repair [84]. In cancer cells which have an acquired resistance to apoptosis [85], this impairment of DSB repair could instead favor error-prone types of end joining (alt-EJ), which fuel cancer progression by exacerbating genomic instability [2]. However, this appealing hypothetical scenario warrants further experimental investigation.

### 2.5. Alterations in Copy Number Variation of the miRNA-hosting FRAXA Regions Differentially Affect Outcomes in Male Versus Female Cancer Patients

It has been proposed that cancer exhibits sexual dimorphism [86]. There are widespread sex differences in tumor incidence, progression, molecular phenotypes, and response to treatment. These differences arise not only by highly gendered human environments (occupations, drug use, etc.), which expose males and females differently to disease risks, but also by gonadal hormones and sex chromosomes (reviewed in [86]). With this in mind, and given that FRAXA is located at the X chromosome, we checked whether alterations in the FRAXA-residing, DSB repair-targeting genes could affect cancer patient outcomes differentially in females versus males.

**Figure 6:**
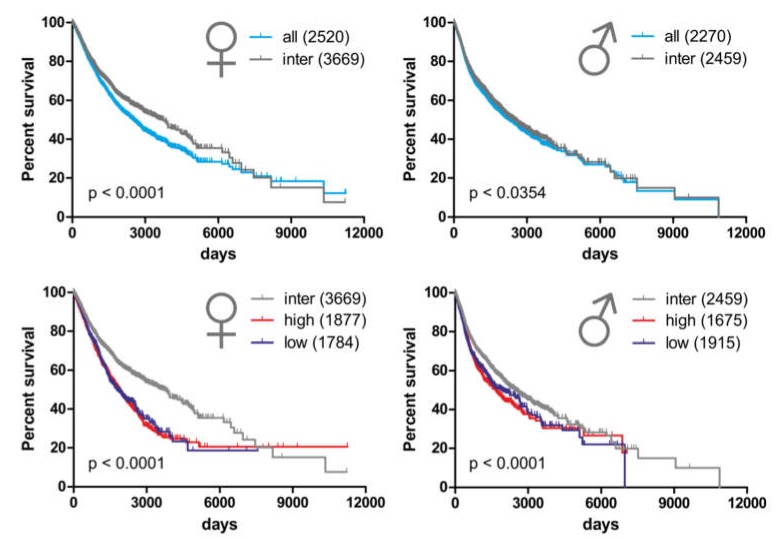
An intact region encompassing the FX-miR clusters is more beneficial to female relative to male patient survival, and females are more susceptible to CNV alterations. Top: Intermediate CNV (inter), representing an intact chromosomal region, has more pronounced effect in female (left) versus male (right) patients, when compared with all females and all males of PanCan cohort, respectively. Bottom: Compared to intermediate CNV, high and low CNVs have a more drastic effect on survival of females (left) relative to males (right). Numbers in parentheses represent median survival in number of days for each condition.

To this end, we investigated whether copy number variations (CNV) in the region encompassing clusters MIR888-892 and MIR506-514 have an impact on survival in male versus female cancer patients. We classified CNVs of this region into low (lower CNV quartile), intermediate or high (upper CNV quartile), where intermediate reflects normal chromosomal region status, while low and high reflect losses or gains of the miRNA genes, correspondingly. First, in PanCan cohort, we compared females and males with intermediate CNVs to the whole female and male population, respectively. We found that females with normal Xq27.3 status have an increased median survival time compared to the whole female population (2520 to 3669 days, 1.46 times). Under the same conditions, the median survival time of males is only marginally improved (2270 to 2459 days, 1.08 times; Figure 6 top).

Then, we checked how high and low CNV status, which reflect chromosomal alterations in this region, modifies survival. We found that in female patients, median survival time for high and low CNVs is reduced by 48.8% and 51.4% (1877 and 1784 days, respectively) compared to females with intermediate CNVs. Notwithstanding, in males, median survival time for high and low CNVs is reduced by 24.6% and 22.1% (1675 and 1915 days, respectively), compared to that of males with intermediate CNVs (Figure 6 bottom). These data collectively imply that a normal Xq27.3 status is linked with better survival, and this effect is more pronounced in females. Alterations (either increases or decreases in CNV) in the region encompassing the MIR506-514 and MIR888-892 clusters shorten median survival time, and females are more vulnerable to these alterations than males. Overall, CNVs in the region encompassing the MIR506-514 and MIR888-892 clusters discriminate the disease outcomes between female and male patients. Given that recent studies underscore sex-biases in mutation load and genomic stability which are associated with genome-wide differences in cancer types other than those affecting reproductive tissues [87], it would be interesting to investigate whether the FX-miR clusters could contribute to these biases through the differential regulation of axes of FRAXA miR/DSB repair targets in male and female patients. A better appreciation of these differences between the two sexes could be of substantial value for cancer prevention and treatment personalization in the future.

## 3. Materials and Methods

### 3.1. Identification of miRNA Genes in Common and Rare Fragile Sites

We used recently published FS [40] and their genomic locations (hg19), and merged them with the data from miRBase (hg19), using the Join intervals function of the Galaxy server [88] to obtain a table of miRNAs residing at those FS. Overlapping FS and bands reported to indiscriminately contain both common and rare fragile sites were excluded from the analysis to reduce any bias. MiRNA density was calculated by dividing the number of miRNAs in each FS by the length of the respective FS. The sum of all miRNAs residing in RFS or CFS was divided by the total length of all RFS or CFS, respectively, to express the ratio of miRNAs/Mbp in RFS versus CFS.

### 3.2. MicroRNA Target Prediction

We screened the microT-CDS database v5.0 [60,61] for miRNAs, that can potentially bind and thereby inhibit the 31 DSB repair factors (Table 1) in both human and mouse. These lists were juxtaposed with the miRNA genes residing in each FS. The number of targets per FS was calculated as number of (non-unique) DSB repair factors that are potentially inhibited by the miRNAs in a FS. ROC curves for the ranking performance of RFS were generated using GraphPad Prism (San Diego, CA, USA).

### 3.3. Kaplan–Meier Survival Analysis of TCGA Patient Data

Patient data of gene expression (GDC-PANCAN.htseq_fpkm-uq), copy number variation (GDC-PANCAN.masked_cnv), miRNA expression (GDC-PANCAN.mirna) and clinical data (GDC-PANCAN.basic_phenotype) were obtained from the TCGA database. Tissue-wise data were extracted from the PanCan data. Cox regression survival as well as Kaplan–Meier analyses were performed in **R**.

### 3.4. Expression Correlation

Expression correlation between miRNAs and genes was calculated in **R** using Spearman correlation.

### 3.5. Statistical Analysis

A statistical analysis was performed using a log rank test. *p* values less than 0.05 were considered as significant.

## 4. Conclusions

Herein, we report a genomic arrangement in RFS, where miRNA genes targeting DSB repair pathways are over-accumulated at FRAXA site, upstream of CGG trinucleotide repeat sequences of the FMR1 gene. Axes of these miRNAs and their corresponding DSB repair gene targets affect survival in a cancer type-specific manner and CNVs in the region encompassing the FX-miR clusters discriminate on survival probability in male and female cancer patients. It would be intriguing to investigate, upon conditions of FRAXA expression in the cell milieu, such as folate insufficiency: (a) if deregulation of these miRNA genes occurs which could activate these axes, and (b) whether there is an interplay between the FX-clusters with their downstream GCCC repeats, to cause wide-spread genomic instability under conditions of FRAXA expression.

## Figures and Tables

**Table 1:** Components and upstream regulators of the DSB repair pathways (HR and NHEJ).

Repair Component	Repair Function	Ref.
**Common/upstream factors**		
ATM	activation of checkpoint signaling, orchestrating cell cycle with DNA repair	[3]
ATR	activation of checkpoint signaling, orchestrating cell cycle with DNA repair	[3]
CHEK1	checkpoint-mediated cell cycle arrest	[4]
CHEK2	checkpoint-mediated cell cycle arrest	[4]
TOPBP1	checkpoint control	[5]
MDC1	checkpoint control	[6]
H2AX/H2afx	DNA double-strand break sensor	[7]
PARP1	DNA double-strand break sensor, recruitment of DNA repair proteins	[8,9]
**Non-Homologous End Joining (NHEJ)**		
ASF1A	facilitates ATM-MDC1 interaction (checkpoint control), recruitment of TP53BP1	[10]
TP53BP1/Trp53bp1	pathway choice: DNA end resection inhibitor	[11,12]
Ku70/Ku80 (XRCC6/5)	DNA-binding, end-protection recruitment of NHEJ complex	[13]
APLF	DNA-end processing, facilitate DNA ligation	[14,15]
Artemis (DCLRE1C)	DNA-end processing	[16]
PRKDC	DNA-end protection and activation of Artemis	[16,17]
LIG4	ligation of DNA ends	[18,19]
XRCC4	facilitates LIG4 activity	[18,19]
NHEJ1 (XLF)	facilitates LIG4 activity	[20,21]
**Homologous recombination (HR)**		
BRCA1	pathway choice: antagonizes TP53BP1 (DNA-end resection activator)	[12]
BRCA2	mediator of strand invasion	[22]
EXO1	pathway choice: DNA-end resection	[23]
PALB2	recruitment and stabilization of BRCA2 and RAD51	[24,25]
RAD50-MRE11-NBN	MRN complex: DNA-end resection	[26]
RAD51	strand invasion and exchange of homologous DNA sequences	[27,28]
RBBP8 (CtIP)	DNA-end resection	[12,29]
SPO11	induction of programmed meiotic DNA double-strand break	[30,31]
BRIP1 (BACH1)	co-factor of BRCA1	[32]
XRCC2	paralog of RAD51, facilitates strand invasion	[33,34]
BLM	pathway choice: DNA-end resection	[23]
RPA	stabilization of ssDNA	[34,35,36]

## Supplementary Materials

The following are available online at https://www.mdpi.com/2072-6694/12/4/876/s1, Table S1: Characterization of fragile site, Table S2: Ranking of fragile sites based on the density of miRNA genes that target DSB repair pathways, Table S3: Ranking of fragile sites based on the density of targeted DSB repair factors by miRNAs, Table S4: DSB repair targets of miRNA genes that reside in mouse and human FRAXA, Table S5: Cox regression results of DSB repair factors on prognosis in PanCan, Table S6: Expression correlation of Fx-miRs and DSB repair factors in PanCan, Table S7: Experimentally validated effects of FX-miRs across cancer types.
