# Supplementary material for: MiRNAs Targeting Double Strand DNA Repair Pathways Lurk in Genomically Unstable Rare Fragile Sites and Determine Cancer Outcomes
Source: Cancers (Basel). 2020 Apr 3;12(4):876. doi: 10.3390/cancers12040876 (PMC7226545; doi:10.3390/cancers12040876)
Supplement: Supplementary file 1 [file cancers-12-00876-s001.zip › submission/Supplementary Files/#Supplementary Table S7.docx]

| **miRNA** | **Cancer type(s)** | **Effect on cancer** | **Cellular process** | **Validated target(s)** | **Study system(s)** | **Ref.** |
| --- | --- | --- | --- | --- | --- | --- |
| **hsa-miR-888** | bladder | oncogenic | DSB repair, cell invasion | APLF | Cancer cell lines, cancer patient samples | [1–4] |
|  | breast | oncogenic | Cell migration, invasion and stemness | nd | Cancer cell lines, mouse xenografts |  |
|  | prostate | oncogenic | Cell proliferation, migration, invasion and colony formation | RBL1, KLF5, TIMP2, SMAD4 | Cancer cell lines, cancer patient samples , mouse xenografts |  |
| **hsa-miR-890** | breast | antioncogenic | Cell proliferation and invasion | CD174 | Cancer cell lines, cancer patient samples | [4–6] |
|  | prostate | antioncogenic | Cell proliferation, migration and invasion, DNA repair, radiosensitivity | MAD2L2, WEE1 | Cancer cell lines, cancer patient samples |  |
| **hsa-miR-891a** | lung | oncogenic | nd | nd | Cancer patient samples | [4,7] |
|  | prostate | oncogenic | Cell proliferation, migration and invasion | nd | Cancer cell lines, cancer patient samples, , mouse xenografts |  |
| **hsa-miR-891b** | breast | antioncogenic | chemosensitivity | PARP1 | Cancer cell lines | [4,8,9] |
|  | prostate | oncogenic | Cell proliferation, migration and invasion | nd | Cancer cell lines, cancer patient samples |  |
|  | Wilms tumor | antioncogenic | Chemosensitivity | nd | Cancer cell lines |  |
| **hsa-miR-892a** | colorectal | oncogenic | Cell proliferation and colony formation | PPP2R2A | Cancer cell lines, cancer patient samples | [4,9,10] |
|  | hepatocellular | oncogenic | Cell proliferation, migration and invasion | CD226 | Cancer cell lines, cancer patient samples |  |
|  | prostate | oncogenic | Cell proliferation, migration and invasion | Nd | Cancer cell lines, cancer patient samples |  |
| **hsa-miR-892b** | Breast | antioncogenic | Cell proliferation and invasion, angiogenesis | TRAF2, TAK1, TAB3 | Cancer cell lines, cancer patient samples , mouse xenografts | [4,11] |
|  | Prostate | oncogenic | Cell proliferation and migration | nd | Cancer cell lines, cancer patient samples |  |
| **hsa-miR-892c** | Prostate | antioncogenic | Cell proliferation and migration | nd | Cancer cell lines, cancer patient samples | [4] |
| **hsa-miR-506** | bladder | antioncogenic | cell proliferation, migration and invasion, EMT | RWDD4 | Cancer cell lines, cancer patient samples | [12–17] |
|  | Breast | antioncogenic | cell migration and invasion, EMT | nd | Cancer cell lines, cancer patient samples |  |
|  | cervical | antioncogenic | Cell proliferation, cell cycle arrest, apoptosis, chemosensitivity | GLI3 | Cancer cell lines, cancer patient samples, mouse xengrafts |  |
|  | Ovarian | antioncogenic | Cell proliferation, senescence, chemosensitivity | CDK4, CDK6, RAD51 | Cancer cell lines, cancer patient samples , mouse xenografts |  |
|  | pancreatic | antioncogenic | Cell proliferation | PIM3 | Cancer cell lines, cancer patient samples |  |
| **hsa-miR-507** | breast | antioncogenic | Cell invasion, chemotaxis, actin polymerization, metastasis | Flt-1 | Cancer cell lines, cancer patient samples , mouse xenografts | [18] |
| **hsa-miR-508** | breast | antioncogenic | Cell invasion, EMT | ZEB1 | Cancer cell lines, cancer patient samples | [19–23] |
|  | gastric | antioncogenic | Cell proliferation, migration and invasion, apoptosis, cell cycle arrest | SKP2 | Cancer cell lines, cancer patient samples |  |
|  | glioma | antioncogenic | Cell proliferation, migration and invasion | nd | Cancer cell lines, cancer patient samples |  |
|  | hepatocellular | antioncogenic | Cell proliferation, migration and invasion | MESDC1 | Cancer cell lines, cancer patient samples , mouse xenografts |  |
|  | ovarian | antioncogenic | cell proliferation, migration and invasion, EMT | MAPK1 | Cancer cell lines, cancer patient samples |  |
| **hsa-miR-509** | cervical | antioncogenic | Cell proliferation, migration and invasion, apoptosis, cell cycle arrest | MDM2 | Cancer cell lines, cancer patient samples | [24–29] |
|  | osteosarcoma | antioncogenic | Cell proliferation, migration and invasion, chemosensitivity | ARHGAP1, AXL, TRIB2 | Cancer cell lines, cancer patient samples |  |
|  | ovarian | antioncogenic | Cell migration and invasion, spheroid formation | YAP1 | Cancer cell lines, cancer patient samples |  |
|  | papillary thyroid carcinoma | oncogenic | Cell proliferation, migration and invasion | SFRP1 | Cancer cell lines, cancer patient samples |  |
|  | renal cell carcinoma | antioncogenic | Cell proliferation, and migration, apoptosis | nd | Cancer cell lines, cancer patient samples |  |
| **hsa-miR-510** | breast | oncogenic | Cell proliferation, migration, invasion and colony formation | PRDX1 | Cancer cell lines,, mouse xenografts | [30–32] |
|  | lung | oncogenic | Cell proliferation and invasion | SRCIN1 | Cancer cell lines, cancer patient samples |  |
|  | ovarian | oncogenic | nd | nd | Cancer patient samples |  |
| **hsa-miR-513a** | breast | oncogenic | Cell resistance to serum starvation | NR3C3 | Cancer cell lines, cancer patient samples | [34–37] |
|  | glioblastoma | oncogenic | chemosensitivity | NEDD4L | Cancer cell lines, cancer patient samples |  |
|  | lung | antioncogenic | Cell migration and invasion, regulation of pro-inflammator environment, chemosensitivity | ITG-β8, GSTP1 | Cancer cell lines |  |
|  | osteosarcoma | antioncogenic | radiosensitivity | APE1 | Cancer cell lines, cancer patient samples |  |
| **hsa-miR-513b** | gastric | antioncogenic | Cell proliferation and migration, apoptosis | HMGB3 | Cancer cell lines, cancer patient samples | [38–40] |
|  | lung | antioncogenic | Cell proliferation, migration and invasion, apoptosis | HMGB3 | Cancer cell lines, cancer patient samples |  |
|  | testicular embryonal carcinoma | antioncogenic | Cell proliferation, apoptosis | IRF2 | Cancer cell lines |  |
| **hsa-miR-513c** | glioblastoma | antioncogenic | Cell proliferation and adhesion | LRP6 | Cancer cell lines, cancer patient samples | [41,42] |
|  | neuroblastoma | antioncogenic | Cell proliferation, migration and invasion | GLS | Cancer cell lines, cancer patient samples |  |
| **hsa-miR-514a** | clear renal cell carcinoma | antioncogenic | Cell proliferation, migration and invasion, EMT | EGFR | Cancer cell lines, cancer patient samples, mouse xenografts | [43,44] |
|  | melanoma | oncogenic | , chemosensitivity | NF1 | Cancer cell lines |  |
| **hsa-miR-514b** | colorectal | antioncogenic/ oncogenic | Cell proliferation, migration and invasion, EMT, chemoresistance | CDH1, CLDN1, FZD, NTN1 | Cancer cell lines,, mouse xenografts (tail veins) | [45] |

*nd - *not determined*

**References**

1. Richter, C.; Marquardt, S.; Li, F.; Spitschak, A.; Murr, N.; Edelhäuser, B.A.H.; Iliakis, G.; Pützer, B.M.; Logotheti, S. Rewiring E2F1 with classical NHEJ via APLF suppression promotes bladder cancer invasiveness. *J. Exp. Clin. Cancer Res.* **2019**, *38*, 292, doi:10.1186/s13046-019-1286-9.

2. Lewis, H.; Lance, R.; Troyer, D.; Beydoun, H.; Hadley, M.; Orians, J.; Benzine, T.; Madric, K.; Semmes, O.J.; Drake, R.; et al. miR-888 is an expressed prostatic secretions-derived microRNA that promotes prostate cell growth and migration. *Cell Cycle* **2014**, *13*, 227–239, doi:10.4161/cc.26984.

3. Huang, S.; Chen, L. MiR-888 regulates side population properties and cancer metastasis in breast cancer cells. *Biochem. Biophys. Res. Commun.* **2014**, *450*, 1234–1240, doi:10.1016/j.bbrc.2014.05.022.

4. Hasegawa, T.; Glavich, G.J.; Pahuski, M.; Short, A.; Semmes, O.J.; Yang, L.; Galkin, V.; Drake, R.; Esquela-Kerscher, A. Characterization and Evidence of the miR-888 Cluster as a Novel Cancer Network in Prostate. *Mol. Cancer Res.* **2018**, *16*, 669–681, doi:10.1158/1541-7786.MCR-17-0321.

5. Wang, C.; Xu, C.; Niu, R.; Hu, G.; Gu, Z.; Zhuang, Z. MiR-890 inhibits proliferation and invasion and induces apoptosis in triple-negative breast cancer cells by targeting CD147. *BMC Cancer* **2019**, *19*, 577, doi:10.1186/s12885-019-5796-9.

6. Hatano, K.; Kumar, B.; Zhang, Y.; Coulter, J.B.; Hedayati, M.; Mears, B.; Ni, X.; Kudrolli, T.A.; Chowdhury, W.H.; Rodriguez, R.; et al. A functional screen identifies miRNAs that inhibit DNA repair and sensitize prostate cancer cells to ionizing radiation. *Nucleic Acids Res.* **2015**, *43*, 4075–4086, doi:10.1093/nar/gkv273.

7. Lee, H.-Y.; Han, S.-S.; Rhee, H.; Park, J.H.; Lee, J.S.; Oh, Y.-M.; Choi, S.S.; Shin, S.-H.; Kim, W.J. Differential expression of microRNAs and their target genes in non-small-cell lung cancer. *Mol. Med. Rep.* **2015**, *11*, 2034–2040, doi:10.3892/mmr.2014.2890.

8. Xu, S.; Zhao, C.; Jia, Z.; Wang, X.; Han, Y.; Yang, Z. Down-regulation of PARP1 by miR-891b sensitizes human breast cancer cells to alkylating chemotherapeutic drugs. *Arch. Gynecol. Obstet.* **2017**, *296*, 543–549, doi:10.1007/s00404-017-4444-3.

9. Jia, B.; Tan, L.; Jin, Z.; Jiao, Y.; Fu, Y.; Liu, Y. MiR-892a Promotes Hepatocellular Carcinoma Cells Proliferation and Invasion Through Targeting CD226. *J. Cell. Biochem.* **2017**, *118*, 1489–1496, doi:10.1002/jcb.25808.

10. Liang, W.-l.; Cao, J.; Xu, B.; Yang, P.; Shen, F.; Sun, Z.; Li, W.-l.; Wang, Q.; Liu, F. miR-892a regulated PPP2R2A expression and promoted cell proliferation of human colorectal cancer cells. *Biomed. Pharmacother.* **2015**, *72*, 119–124, doi:10.1016/j.biopha.2015.04.015.

11. Jiang, L.; Yu, L.; Zhang, X.; Lei, F.; Wang, L.; Liu, X.; Wu, S.; Zhu, J.; Wu, G.; Cao, L.; et al. miR-892b Silencing Activates NF-κB and Promotes Aggressiveness in Breast Cancer. *Cancer Res.* **2016**, *76*, 1101–1111, doi:10.1158/0008-5472.CAN-15-1770.

12. Wen, S.-Y.; Lin, Y.; Yu, Y.-Q.; Cao, S.-J.; Zhang, R.; Yang, X.-M.; Li, J.; Zhang, Y.-L.; Wang, Y.-H.; Ma, M.-Z.; et al. miR-506 acts as a tumor suppressor by directly targeting the hedgehog pathway transcription factor Gli3 in human cervical cancer. *Oncogene* **2015**, *34*, 717–725, doi:10.1038/onc.2014.9.

13. Du, J.; Zheng, X.; Cai, S.; Zhu, Z.; Tan, J.; Hu, B.; Huang, Z.; Jiao, H. MicroRNA‑506 participates in pancreatic cancer pathogenesis by targeting PIM3. *Mol. Med. Rep.* **2015**, *12*, 5121–5126, doi:10.3892/mmr.2015.4109.

14. Liu, G.; Sun, Y.; Ji, P.; Li, X.; Cogdell, D.; Yang, D.; Parker Kerrigan, B.C.; Shmulevich, I.; Chen, K.; Sood, A.K.; et al. MiR-506 suppresses proliferation and induces senescence by directly targeting the CDK4/6-FOXM1 axis in ovarian cancer. *J. Pathol.* **2014**, *233*, 308–318, doi:10.1002/path.4348.

15. Arora, H.; Qureshi, R.; Park, W.-Y. miR-506 regulates epithelial mesenchymal transition in breast cancer cell lines. *PLoS ONE* **2013**, *8*, e64273, doi:10.1371/journal.pone.0064273.

16. Hou, Y. MiR-506 inhibits cell proliferation, invasion, migration and epithelial-to-mesenchymal transition through targeting RWDD4 in human bladder cancer. *Oncol. Lett.* **2019**, *17*, 73–78, doi:10.3892/ol.2018.9594.

17. Liu, G.; Yang, D.; Rupaimoole, R.; Pecot, C.V.; Sun, Y.; Mangala, L.S.; Li, X.; Ji, P.; Cogdell, D.; Hu, L.; et al. Augmentation of response to chemotherapy by microRNA-506 through regulation of RAD51 in serous ovarian cancers. *J. Natl. Cancer Inst.* **2015**, *107*, doi:10.1093/jnci/djv108.

18. Jia, L.; Liu, W.; Cao, B.; Li, H.; Yin, C. MiR-507 inhibits the migration and invasion of human breastcancer cells through Flt-1 suppression. *Oncotarget* **2016**, *7*, 36743–36754, doi:10.18632/oncotarget.9163.

19. Guo, S.-J.; Zeng, H.-X.; Huang, P.; Wang, S.; Xie, C.-H.; Li, S.-J. MiR-508-3p inhibits cell invasion and epithelial-mesenchymal transition by targeting ZEB1 in triple-negative breast cancer. *Eur. Rev. Med. Pharmacol. Sci.* **2018**, *22*, 6379–6385, doi:10.26355/eurrev_201810_16050.

20. Hong, L.; Wang, Y.; Chen, W.; Yang, S. MicroRNA-508 suppresses epithelial-mesenchymal transition, migration, and invasion of ovarian cancer cells through the MAPK1/ERK signaling pathway. *J. Cell. Biochem.* **2018**, *119*, 7431–7440, doi:10.1002/jcb.27052.

21. Duan, X.; Bai, J.; Wei, J.; Li, Z.; Liu, X.; Xu, G. MicroRNA-508-5p suppresses metastasis in human gastric cancer by targeting S-phase kinase‑associated protein 2. *Mol. Med. Rep.* **2017**, *16*, 2163–2171, doi:10.3892/mmr.2017.6793.

22. Wu, S.G.; Huang, Y.J.; Bao, B.; Wu, L.M.; Dong, J.; Liu, X.H.; Li, Z.H.; Wang, X.Y.; Wang, L.; Chen, B.J.; et al. miR-508-5p acts as an anti-oncogene by targeting MESDC1 in hepatocellular carcinoma. *Neoplasma* **2017**, *64*, 40–47, doi:10.4149/neo_2017_105.

23. Liu, Y.-H.; Li, B.; Meng, F.-G.; Qiu, L. MiR-508-5p is a prognostic marker and inhibits cell proliferation and migration in glioma. *Eur. Rev. Med. Pharmacol. Sci.* **2017**, *21*, 76–81.

24. Yang, C.; Wang, Y.; Yang, W.; Gao, Y.; Zhao, B.; Yang, X. MiR-509-5p improves the proliferative and invasive abilities of papillary thyroid carcinoma cells by inhibiting SFRP1. *Arch. Med. Sci.* **2019**, *15*, 968–978, doi:10.5114/aoms.2019.85904.

25. Patil, S.L.; Palat, A.; Pan, Y.; Rajapakshe, K.; Mirchandani, R.; Bondesson, M.; Yustein, J.T.; Coarfa, C.; Gunaratne, P.H. MicroRNA-509-3p inhibits cellular migration, invasion, and proliferation, and sensitizes osteosarcoma to cisplatin. *Sci. Rep.* **2019**, *9*, 19089, doi:10.1038/s41598-019-55170-2.

26. Guo, J.; Wu, Q.; Peng, X.; Yu, B. miR-509-5p Inhibits the Proliferation and Invasion of Osteosarcoma by Targeting TRIB2. *Biomed Res. Int.* **2019**, *2019*, 2523032, doi:10.1155/2019/2523032.

27. Pan, Y.; Robertson, G.; Pedersen, L.; Lim, E.; Hernandez-Herrera, A.; Rowat, A.C.; Patil, S.L.; Chan, C.K.; Wen, Y.; Zhang, X.; et al. miR-509-3p is clinically significant and strongly attenuates cellular migration and multi-cellular spheroids in ovarian cancer. *Oncotarget* **2016**, *7*, 25930–25948, doi:10.18632/oncotarget.8412.

28. Zhang, W.-B.; Pan, Z.-Q.; Yang, Q.-S.; Zheng, X.-M. Tumor suppressive miR-509-5p contributes to cell migration, proliferation and antiapoptosis in renal cell carcinoma. *Ir. J. Med. Sci.* **2013**, *182*, 621–627, doi:10.1007/s11845-013-0941-y.

29. Ren, Z.-J.; Nong, X.-Y.; Lv, Y.-R.; Sun, H.-H.; An, P.-P.; Wang, F.; Li, X.; Liu, M.; Tang, H. Mir-509-5p joins the Mdm2/p53 feedback loop and regulates cancer cell growth. *Cell Death Dis.* **2014**, *5*, e1387, doi:10.1038/cddis.2014.327.

30. Guo, Q.J.; Mills, J.N.; Bandurraga, S.G.; Nogueira, L.M.; Mason, N.J.; Camp, E.R.; Larue, A.C.; Turner, D.P.; Findlay, V.J. MicroRNA-510 promotes cell and tumor growth by targeting peroxiredoxin1 in breast cancer. *Breast Cancer Res.* **2013**, *15*, R70, doi:10.1186/bcr3464.

31. Wu, W.; He, L.; Huang, Y.; Hou, L.; Zhang, W.; Zhang, L.; Wu, C. MicroRNA-510 Plays Oncogenic Roles in Non-Small Cell Lung Cancer by Directly Targeting SRC Kinase Signaling Inhibitor 1. *Oncol. Res.* **2019**, *27*, 879–887, doi:10.3727/096504018X15451308507747.

32. Zhang, X.; Guo, G.; Wang, G.; Zhao, J.; Wang, B.; Yu, X.; Ding, Y. Profile of differentially expressed miRNAs in high-grade serous carcinoma and clear cell ovarian carcinoma, and the expression of miR-510 in ovarian carcinoma. *Mol. Med. Rep.* **2015**, *12*, 8021–8031, doi:10.3892/mmr.2015.4485.

33. Li, H.; Huang, D.; Hang, S. Salidroside inhibits the growth, migration and invasion of Wilms' tumor cells through down-regulation of miR-891b. *Life Sci.* **2019**, *222*, 60–68, doi:10.1016/j.lfs.2019.02.052.

34. Dai, N.; Qing, Y.; Cun, Y.; Zhong, Z.; Li, C.; Zhang, S.; Shan, J.; Yang, X.; Dai, X.; Cheng, Y.; et al. miR-513a-5p regulates radiosensitivity of osteosarcoma by targeting human apurinic/apyrimidinic endonuclease. *Oncotarget* **2018**, *9*, 25414–25426, doi:10.18632/oncotarget.11003.

35. Muti, P.; Donzelli, S.; Sacconi, A.; Hossain, A.; Ganci, F.; Frixa, T.; Sieri, S.; Krogh, V.; Berrino, F.; Biagioni, F.; et al. MiRNA-513a-5p inhibits progesterone receptor expression and constitutes a risk factor for breast cancer: the hOrmone and Diet in the ETiology of breast cancer prospective study. *Carcinogenesis* **2018**, *39*, 98–108, doi:10.1093/carcin/bgx126.

36. Chen, K.-C.; Chen, P.-H.; Ho, K.-H.; Shih, C.-M.; Chou, C.-M.; Cheng, C.-H.; Lee, C.-C. IGF-1-enhanced miR-513a-5p signaling desensitizes glioma cells to temozolomide by targeting the NEDD4L-inhibited Wnt/β-catenin pathway. *PLoS ONE* **2019**, *14*, e0225913, doi:10.1371/journal.pone.0225913.

37. da Silveira, M.B.; Lima, K.F.; da Silva, A.R.; Dos Santos, R.A.S.; Moraes, K.C.M. Mir-513a-3p contributes to the controlling of cellular migration processes in the A549 lung tumor cells by modulating integrin β-8 expression. *Mol. Cell. Biochem.* **2018**, *444*, 43–52, doi:10.1007/s11010-017-3229-0.

38. Wang, J.; Sheng, Z.; Cai, Y. Effects of microRNA-513b on cell proliferation, apoptosis, invasion, and migration by targeting HMGB3 through regulation of mTOR signaling pathway in non-small-cell lung cancer. *J. Cell. Physiol.* **2019**, *234*, 10934–10941, doi:10.1002/jcp.27921.

39. Chen, X.; Zhao, G.; Wang, F.; Gao, F.; Luo, H.; Wang, Y.; Du, Y.; Chen, X.; Xue, C.; Dong, Z.; et al. Upregulation of miR-513b inhibits cell proliferation, migration, and promotes apoptosis by targeting high mobility group-box 3 protein in gastric cancer. *Tumour Biol.* **2014**, *35*, 11081–11089, doi:10.1007/s13277-014-2405-z.

40. Wang, X.; Zhang, X.; Wang, G.; Wang, L.; Lin, Y.; Sun, F. Hsa-miR-513b-5p suppresses cell proliferation and promotes P53 expression by targeting IRF2 in testicular embryonal carcinoma cells. *Gene* **2017**, *626*, 344–353, doi:10.1016/j.gene.2017.05.033.

41. Xia, H.-L.; Lv, Y.; Xu, C.-W.; Fu, M.-C.; Zhang, T.; Yan, X.-M.; Dai, S.; Xiong, Q.-W.; Zhou, Y.; Wang, J.; et al. MiR-513c suppresses neuroblastoma cell migration, invasion, and proliferation through direct targeting glutaminase (GLS). *Cancer Biomark.* **2017**, *20*, 589–596, doi:10.3233/CBM-170577.

42. Xu, J.; Sun, T.; Hu, X. microRNA-513c suppresses the proliferation of human glioblastoma cells by repressing low-density lipoprotein receptor-related protein 6. *Mol. Med. Rep.* **2015**, *12*, 4403–4409, doi:10.3892/mmr.2015.3913.

43. Ke, X.; Zeng, X.; Wei, X.; Shen, Y.; Gan, J.; Tang, H.; Hu, Z. MiR-514a-3p inhibits cell proliferation and epithelial-mesenchymal transition by targeting EGFR in clear cell renal cell carcinoma. *Am. J. Transl. Res.* **2017**, *9*, 5332–5346.

44. Stark, M.S.; Bonazzi, V.F.; Boyle, G.M.; Palmer, J.M.; Symmons, J.; Lanagan, C.M.; Schmidt, C.W.; Herington, A.C.; Ballotti, R.; Pollock, P.M.; et al. miR-514a regulates the tumour suppressor NF1 and modulates BRAFi sensitivity in melanoma. *Oncotarget* **2015**, *6*, 17753–17763, doi:10.18632/oncotarget.3924.

45. Ren, L.-L.; Yan, T.-T.; Shen, C.-Q.; Tang, J.-Y.; Kong, X.; Wang, Y.-C.; Chen, J.; Liu, Q.; He, J.; Zhong, M.; et al. The distinct role of strand-specific miR-514b-3p and miR-514b-5p in colorectal cancer metastasis. *Cell Death Dis.* **2018**, *9*, 687, doi:10.1038/s41419-018-0732-5.
